# Combined Evidence Annotation of Transposable Elements in Genome Sequences

**DOI:** 10.1371/journal.pcbi.0010022

**Published:** 2005-07-29

**Authors:** Hadi Quesneville, Casey M Bergman, Olivier Andrieu, Delphine Autard, Danielle Nouaud, Michael Ashburner, Dominique Anxolabehere

**Affiliations:** 1 Laboratoire Dynamique du Génome et Evolution, Institut Jacques Monod, Paris, France; 2 Department of Genetics, University of Cambridge, Cambridge, United Kingdom; Washington University in St. Louis, United States of America

## Abstract

Transposable elements (TEs) are mobile, repetitive sequences that make up significant fractions of metazoan genomes. Despite their near ubiquity and importance in genome and chromosome biology, most efforts to annotate TEs in genome sequences rely on the results of a single computational program, RepeatMasker. In contrast, recent advances in gene annotation indicate that high-quality gene models can be produced from combining multiple independent sources of computational evidence. To elevate the quality of TE annotations to a level comparable to that of gene models, we have developed a combined evidence-model TE annotation pipeline, analogous to systems used for gene annotation, by integrating results from multiple homology-based and de novo TE identification methods. As proof of principle, we have annotated “TE models” in *Drosophila melanogaster* Release 4 genomic sequences using the combined computational evidence derived from RepeatMasker, BLASTER, TBLASTX, all-by-all BLASTN, RECON, TE-HMM and the previous Release 3.1 annotation. Our system is designed for use with the Apollo genome annotation tool, allowing automatic results to be curated manually to produce reliable annotations. The euchromatic TE fraction of *D. melanogaster* is now estimated at 5.3% (cf. 3.86% in Release 3.1), and we found a substantially higher number of TEs (*n* = 6,013) than previously identified (*n* = 1,572). Most of the new TEs derive from small fragments of a few hundred nucleotides long and highly abundant families not previously annotated (e.g., *INE-1*). We also estimated that 518 TE copies (8.6%) are inserted into at least one other TE, forming a nest of elements. The pipeline allows rapid and thorough annotation of even the most complex TE models, including highly deleted and/or nested elements such as those often found in heterochromatic sequences. Our pipeline can be easily adapted to other genome sequences, such as those of the *D. melanogaster* heterochromatin or other species in the genus *Drosophila*.

## Synopsis

A first step in adding value to the large-scale DNA sequences generated by genome projects is the process of annotation—marking biological features on the raw string of adenines, cytosines, guanines, and thymines. The predominant goal in genome annotation thus far has been to identify gene sequences that encode proteins; however, many functional sequences exist in non-protein-coding regions and their annotation remains incomplete. Mobile, repetitive DNA segments known as transposable elements (TEs) are one class of functional sequence in non-protein-coding regions, which can make up large fractions of genome sequences (e.g., about 45% in the human) and can play important roles in gene and chromosome structure and regulation. As a consequence, there has been increasing interest in the computational identification of TEs in genome sequences. Borrowing current ideas from the field of gene annotation, the authors have developed a pipeline to predict TEs in genome sequences that combines multiple sources of evidence from different computational methods. The authors' combined-evidence pipeline represents an important step towards raising the standards of TE annotation to the same quality as that of genes, and should help catalyze their understanding of the biological role of these fascinating sequences.

## Introduction

Transposable elements (TEs) are mobile, repetitive DNA sequences that constitute a structurally dynamic component of genomes. The taxonomic distribution of TEs is virtually ubiquitous: they have been found in nearly all eukaryotic organisms studied, with few exceptions. TEs represent quantitatively important components of genome sequences (e.g., 44.4% of the human genome; [1]), and there is no doubt that modern genomic DNA has evolved in close association with TEs. TEs show high species specificity, and the number and types of TE can differ quite dramatically between even closely related organisms. There is abundant circumstantial evidence that TEs may transfer horizontally between species by mechanisms that remain obscure. The forces controlling the dynamics of TE spread within a species are also poorly understood, as are the systemic effects of the elements on their host genomes. Insertions of individual TEs may lead to genome restructuring (e.g., the occurrence of inversions), mutations in genes, or changes in gene regulation. Some TE insertions may even have become domesticated to play roles in the normal functions of the host (see [2] for review). Despite their manifold effects, abundance, and ubiquity, we understand very little about most aspects of TE biology.

One way of furthering our knowledge of TE biology is through the computational analysis of TEs in the growing number of complete genomic sequences. By detailed comparison of the abundance and distribution of TEs in entire genomes, we can infer the fundamental biological properties of TEs that are shared or that differ among species. However, meaningful inferences about TE biology based on computationally derived TE annotations can only be done if we are confident about the results of these analyses. The hallmark of a strong result in computational biology should be its robustness to the particular method used. The annotation of TEs, however, typically relies on the results of a single computational program, RepeatMasker (http://www.repeatmasker.org/), which recent studies indicate may be “neither the most efficient nor the most sensitive approach” for TE annotation [3]. By contrast, recent advances in the field of gene annotation indicate that high-quality gene models can be produced by combining multiple independent sources of computational evidence [4–9]. With the recent development of several new methods for TE and repeat detection [10–16], it is now possible to apply a similar “combined evidence” approach to elevate the quality of TE annotations to a level comparable to that of gene models.

To achieve this aim, we have developed a TE annotation pipeline that integrates results from multiple homology-based and de novo TE identification methods. Currently, our pipeline uses the combined computational evidence derived from RepeatMasker (http://www.repeatmasker.org/), BLASTER [13], TBLASTX [17], all-by-all BLASTN [17], RECON [10], TE-HMM [14], and previously published TE annotations [18]. We have designed our system to use an “evidence-model” framework and the Apollo genome annotation tool [19], allowing computational evidence to be manually curated in an efficient manner to produce reliable “TE models”. The pipeline allows rapid and thorough annotation of complex TE models, providing key structural details that allow insights into the origin of highly deleted and/or nested elements. In contrast to simply masking repeats, our method provides the means to a complete and accurate annotation of TEs, supported by multiple sources of computational evidence, a goal that has important implications for experimental studies of genome and chromosome biology.

As a test case we have chosen to annotate the euchromatic genomic sequence of the fruit fly, *Drosophila melanogaster*. The 116.8-Mb Release 3 genome sequence of *D. melanogaster* is among the highest quality genome sequences and is a particularly well suited sequence for genome-wide studies of TEs, since repetitive DNA sequences have been finished to high quality and systematically verified by restriction fingerprint analysis [20]. Moreover, the Release 3.1 annotation of *D. melanogaster* includes a manually curated set of TE annotations [18] that can be used as a benchmark for developing and refining TE annotation methodologies. Controlled tests performed here on the Release 3 sequence show that a combined-evidence approach has superior performance over individual TE detection methods, and that a substantially larger fraction of the genome is composed of TEs than previously estimated. We have applied our pipeline to the new 118.4-Mb Release 4 sequence (http://www.fruitfly.org/annot/release4.html), which has closed several of the gaps in Release 3 and has extended the sequence of the pericentomeric regions, to produce a systematic re-annotation of TEs in the *D. melanogaster* genome. The euchromatic TE fraction is now estimated at 5.3% (cf. 3.86% in Release 3.1), and we found a substantially higher number of TEs (*n* = 6,013) than previously identified (*n* = 1,572). We also estimated that 518 TE copies (8.6%) are inserted into at least one other TE, forming a nest of elements. Our pipeline can be easily adapted to other genome sequences, and could markedly increase the efficiency of annotating genomic regions with complex or abundant TE insertions such as heterochromatic sequences.

## Results

### Evaluation of Methods

The first step in the development of our pipeline was to evaluate the abilities of different computational tools that are available to annotate TEs in order to assess the strengths and weaknesses of each method. To do this we re-annotated the *D. melanogaster* Release 3 sequence using different TE detection methods and compared these results to the FlyBase Release 3.1 annotation (http://www.flybase.org/annot/release3.html), which includes the results of a manually curated set of TE annotations published previously by Kaminker et al. [18].

Methods for TE annotation fall into two general classes: (i) methods designed for the annotation of known TE families, which utilize a specific reference sequence (also called a canonical sequence) and (ii) de novo methods designed for the annotation of anonymous TE families, for which no reference sequence has yet been identified. This distinction is necessary since it determines the relevant measures to evaluate different methods for TE detection.

### Methods for the Annotation of Known TE Families

To allow direct comparison with previous results [18], we used the Release 3 genomic sequence as a query to be scanned for similarity to reference sequences in version 7.1 of the Berkeley *Drosophila* Genome Project (BDGP) TE dataset (http://www.fruitfly.org/p_disrupt/TE.html), the same version that was used for the Release 3.1 FlyBase annotation. We initially tested three methods for TE prediction (see Materials and Methods for details): (i) BLASTER using BLASTN followed by chaining with MATCHER (BLRn), (ii) RepeatMasker using default parameters (RM), and (iii) RM using default parameters followed by chaining with MATCHER (RMm). The last method was used to test the benefit of the “chaining algorithm” implemented in MATCHER.

We compared predictions to annotations by calculating sensitivity and specificity values for the number of nucleotides of TE sequence predicted by a method that overlapped (or did not overlap) TEs in the Release 3.1 FlyBase annotation (see Materials and Methods). Note that the computation of specificity is biased here, since it assumes that all TEs in the Release 3.1 FlyBase annotation are known, which is certainly not true. We also compared different categories of overlap between prediction and annotation boundaries to gain deeper insight into the details of TE detection methods (see Materials and Methods). These results are summarized in Table 1.

**Table 1:**
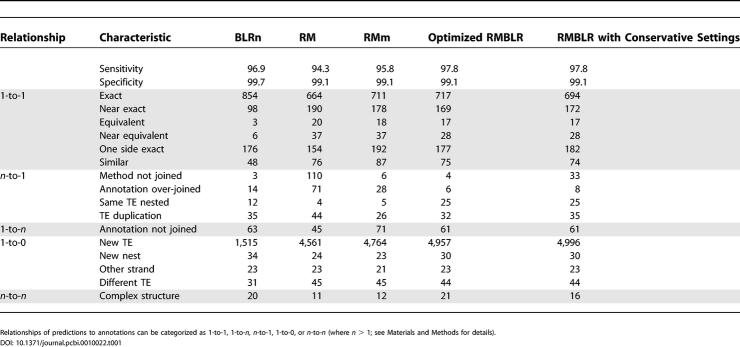
Results of Comparisons between TE Prediction Methods That Use Reference Sequences and the Release 3 FlyBase TE Annotations

Relationships of predictions to annotations can be categorized as 1-to-1, 1-to-*n,* *n*-to-1, 1-to-0, or *n*-to-*n* (where *n* > 1; see Materials and Methods for details).

We found that both the sensitivity and the specificity to predict Release 3.1 TEs were higher for BLRn (96.9% and 99.7%, respectively) than for RM (94.3% and 99.1%, respectively). In addition, 28% more Release 3.1 TEs were predicted exactly by BLRn (*n* = 854) than by RM (*n* = 664). BLRn also made well over an order of magnitude fewer “method not joined” errors (*n* = 3) than RM (*n* = 110), indicating that the BLRn strategy makes high-quality automatic decisions about joining fragments of TEs. RMm had intermediate performance with respect to RM and BLRn for exactly predicting Release 3.1 annotations (*n* = 711), but, like BLRn, had few “method not joined” errors (*n* = 6). These results may be explained partly by the fact the Release 3.1 annotation was produced using BLAST-based methods [18], and that the local alignment stop criterion significantly differs between the BLAST algorithm and the Smith and Waterman algorithm used by RM (in the final search phase). Thus, the good performance of BLRn for predicting Release 3.1 TE boundaries could result from the fact that the same local alignment stop criterion was used. However, differences in local alignment matching cannot explain these results entirely, since RMm outperformed RM to recover exact matches, indicating that the chaining algorithm implemented in MATCHER is a significant improvement over raw RM results for predicting Release 3.1 TE annotations.

RM identified approximately 3-fold more new TEs than BLRn, and thus appears to be a more sensitive method for the detection of previously unannotated TEs. But here also RMm had a better performance for detecting new TEs than RM, so the effects of chaining can also improve RM in this regard. The putative TEs predicted by RM in general were short, as can be seen by the relatively limited effect that an additional 3,000+ predictions had on the genome-wide specificity of RM and RMm.

Given the different performance of these approaches, we developed and tested a fourth strategy that attempts to capitalize on the strengths of both RM and BLRn. This method, called RepeatMasker-BLASTER (RMBLR), combined hits from both BLRn and RM and gave them to MATCHER for chaining. To do this, we normalized alignment scores from BLRn and RM to be the hit length for chaining. As shown in Table 1, an optimized RMBLR had higher sensitivity than RM, RMm, or BLRn alone, produced the highest number of putative new TE annotations, and otherwise retained performance features similar to RMm and BLRn. These results show that a combined approach to TE annotation is more efficient at both recovering known TE annotations and predicting new ones than each method alone.

The results shown in Table 1 also suggest that there were errors in the Release 3.1 FlyBase annotation. Among them, the tools predicted cases where two annotations could be joined automatically (category “annotation not joined” in Table 1) and others where an annotation might be split (category “annotation over-joined” in Table 1). Using the Apollo annotation editor [19] to inspect these errors visually, we found that the fragmented and the nested structures of TEs often could be recovered better with these tools than in the Release 3.1 FlyBase annotation. In addition, using Apollo we found that the many new copies appear to be bona fide remnants of TEs missing from the previous annotation; however, a detailed analysis of Release 4 revealed that many of these new TEs may result from spurious hits to simple repeats in the reference sequence (see below).

### Methods for the Annotation of Anonymous TE Families

We also tested de novo methods to predict TEs that do not use a specific reference sequence, and evaluated the ability of these methods to find TEs in the Release 3.1 *D. melanogaster* annotation. These results serve to determine the ability of each method to identify anonymous TEs, and are important for the annotation of genome sequences where a manually curated reference set of TEs is not available. Individually, we found that these methods have lower performance than those that use specific reference sequences, but together they provide additional evidence that can be used to evaluate TE models in the final manual curation step.

TEs have been predicted anonymously using four different methods: (i) an all-by-all genome comparison with BLASTER using BLASTN followed by chaining with MATCHER and grouping with GROUPER (BLRa), (ii) RECON, using default parameters, (iii) BLASTER using TBLASTX with the entire Repbase Update as the database, followed by chaining with MATCHER (BLRtx), and (iv) a hidden Markov model that detects TE sequences based on nucleotide composition (TE-HMM). Note that for BLRa, we compared coordinates of the group of sequences obtained by GROUPER with a coverage of zero (i.e., all overlapping matches were merged; see Materials and Methods for details).

As above, sensitivity, specificity, and the comparison of boundaries between predictions and annotations were used to evaluate the performance of each method. Note again that, as previously, specificity is here biased because it assumes for its computation that all TEs in the genome are known. Here, specificity may be less meaningful than above, since the ability of these methods to detect new TEs is enhanced, and methods detecting many new TEs would have a correspondingly low specificity. Therefore, we must be careful to interpret specificity here as the ability to detect only already known TEs.

Table 2 shows that all de novo methods had relatively high overall specificity (>88%) to detect Release 3.1 TE annotations, but that RECON gave the best performance to recover Release 3.1 TEs exactly. BLRtx had the highest overall sensitivity to detect Release 3.1 TEs (97.2%), which may be explained by the fact that this method uses Repbase Update, which includes most of the *Drosophila* TEs. This can be shown by a similar analysis with *Drosophila* TEs removed from the Repbase Update (see BLRtxNoDros in Table 2), which gave lower sensitivity (44.2%), fewer new TEs (*n* = 8,110), and no “exact”, “near exact”, or “equivalent” cases. BLRtx and TE-HMM detected thousands more new putative TEs than RECON, BLRa, and the other methods detailed in Table 1, indicating that many new TE families may remain to be described in the *D. melanogaster* genome [13]. These new families are probably low in copy number and represented by nonoverlapping fragments, as suggested by the smaller number of new TEs found by BLRa and RECON. In fact RECON could only detect TEs that are repeated and have copies that are more or less well conserved to their extremities. BLRtx and TE-HMM would be able to detect TEs in few copies (even unique elements) that could be highly diverged and/or degenerate. It is perhaps surprising that BLRtx predicts the highest number of new TEs, since TE-HMM would be able to detect copies for which no distant TE reference sequence is known. However, the high number of BLRtx and BLRtxNoDros predictions may result from an under-joining of fragments of the same TE, as suggested by the large number of “method not joined” cases: *n* = 1,172 (BLRtx) and *n* = 3,587 (BLRtxNoDros). In contrast, the high number of predictions resulting from TE-HMM do not appear to result from under-joining (“method not joined”; *n* = 42), but rather (with their relatively low sensitivity and specificity) suggest a tendency to overpredict using the current parameters. Together these results demonstrate that de novo methods provide specific evidence that can be used to support TE models, but additional development is necessary to fine-tune these approaches to generate accurate TE annotations directly.

**Table 2:**
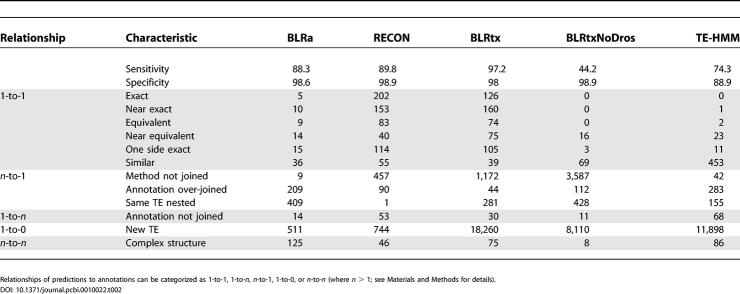
Results of Comparisons between TE Prediction Methods That Do Not Use Reference Sequences and the Release 3 FlyBase TE Annotations

Relationships of predictions to annotations can be categorized as 1-to-1, 1-to-*n,* *n*-to-1, 1-to-0, or *n*-to-*n* (where *n* > 1; see Materials and Methods for details).

### The Annotation Pipeline

**Figure 1:**
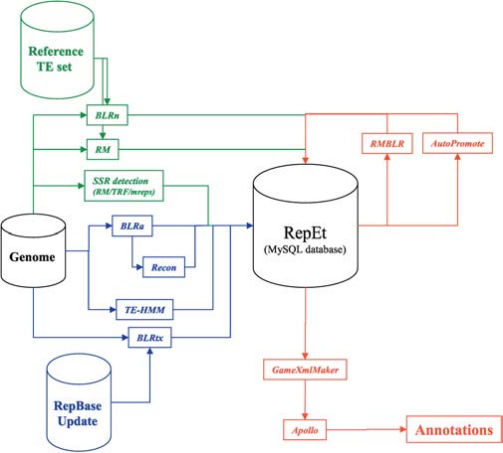
Schematic of Our TE Annotation Pipeline The pipeline is composed of (i) known TE family detection methods such as BLRn, RM, and RMBLR; (ii) satellite detection software such as RM, TRF, and Mreps; (iii) anonymous TE detection methods such BLRa, TE-HMM, RECON, and BLRtx; and (iv) a MySQL database called REPET to manage the results and the annotations. GAME-XML files are then generated from the results stored in the database and loaded into the Apollo genome annotation tool, allowing automatic results to be manually curated to produce a reliable annotation. To facilitate manual curation, we automatically promoted RMBLR results to be the candidate annotation.

Based on these results, we designed an integrated pipeline to compute and store evidence and TE annotations for genome sequences (Figure 1). Our annotation pipeline is composed of (i) TE detection software such as BLASTER, RepeatMasker, TE-HMM, and RECON; (ii) satellite detection software such as RepeatMasker, Tandem Repeat Finder (TRF) [21], and Mreps [22], (iii) a MySQL database (http://www.mysql.com/) to manage the results of these methods and the annotations generated from them; and (iv) Open Portable Batch System (http://www.openpbs.org/) for distributing jobs on a computer cluster. The flexible architecture of this system easily allows other methods for TE detection to be added to this pipeline in the future.

To save computer time and reduce software memory requirements, we segmented the Release 4 genomic sequences into chunks of 200 kb overlapping by 10 kb. Each chunk was then independently analyzed by the different analysis programs, and the results were stored in the MySQL database. GAME-XML (http://www.fruitfly.org/annot/apollo/game.rng.txt) files were then generated from the results stored in the database and loaded into the Apollo genome annotation tool, allowing automatic results to be manually curated to produce a reliable annotation. For this curation we used as evidence tiers (i) the Release 3.1 FlyBase annotations with coordinates mapped to the Release 4 sequences, (ii) BLRn, RM, and RMBLR results using version 9.0 of the BDGP TE reference set, (iii) BLRtx using Repbase Update 8.12, and (iv) RECON, BLRa, and TE-HMM (see Materials and Methods for details). We required all annotations to be supported by at least one of the methods for detecting known TEs—BLRn, RM, or RMBLR. We did not include annotations based solely on anonymous prediction methods since these methods potentially suffer from high false positive rates (Table 2), even though our analyses on Release 3 suggested that there may be additional families of TEs yet to be discovered in the *D. melanogaster* genome. We note that our pipeline is currently designed with the goal of achieving the best possible annotation set of known TEs in a genome sequence, not the discovery of new TE families, an important endeavor in its own right but outside the scope of the current work.

**Figure 2:**
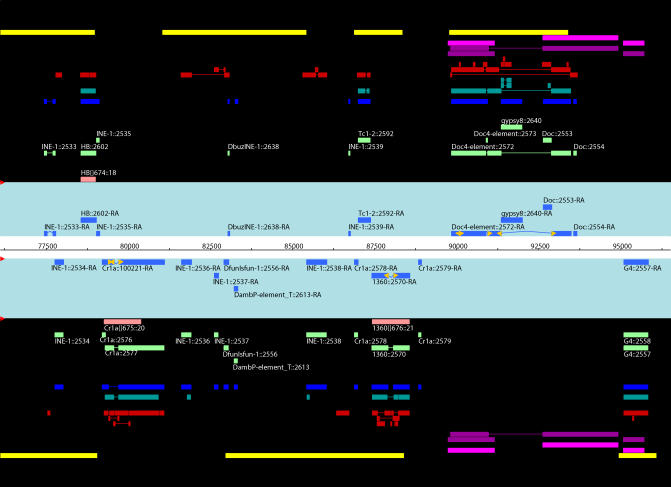
Screenshot of an Apollo View for a Peri-Centromeric Region with Extreme TE Density Curated annotations on both forward strand (top) and reverse strand (bottom) are displayed in the light blue panels. Evidence tiers are shown in the black panels: TE-HMM (yellow), RECON (light purple), BLRa (violet), BLRtx (red), BLRn (teal), RM (blue), RMBLR (light green), and Release 3.1 FlyBase annotations (peach).

To facilitate manual curation, we automatically promoted the results of RMBLR to be the candidate annotation (defined as a set of one or more joined fragments), which could then be validated or modified by the curator in Apollo according to the evidence available in the GAME-XML file (see Figure 2 for an example). In addition, we generated a candidate list of mis-joined matches that were contiguous but not joined by MATCHER because of the size of the deletion or the insertion in the genomic sequence. This list identified potential problem cases to be considered carefully for manual joins in Apollo. Moreover, we used RMBLR with conservative settings (gap penalty of 0.05), intentionally under-joining contiguous matches compared to the optimal setting (gap penalty of 0.04; see Table 1). Hence, the join decision of the most difficult cases is left to the curator. Another consequence of this conservative approach is that only a few annotations were manually split. This happened when two small and distant fragments (generally neighboring copies of *INE-1* [23]) were automatically joined, and the insert between the two fragments did not correspond to another TE (as would be the case for a nested TE). We considered these joins excessive because of the lack of knowledge about the biology of the *INE-1* TE family, for which it is difficult to find a reliable reference sequence. We initially split the five major chromosome arms among five curators for a first-pass manual curation, which was completed in less than 2 wk. Subsequent to this, a single curator performed a second-pass manual curation in order to improve the consistency of manual edit decisions. We examined 10,348 annotations, and only 523 (5%) of them needed to be edited. Finally, we obtained 9,053 unique TE annotations after merging annotations in the overlaps between chunks.

During the manual curation step, we encountered an unexpectedly large number of apparently spurious hits to particular TE families resulting from similarity to simple repeats present in the reference sequence. For example, 236 of 373 predicted TEs for the *roo* family [24] were generated only by matches to the [CA(A/G)]*_n_* repeat in the *roo* reference sequence. Since the number of spurious hits resulting from simple repeats is potentially quite large, we considered several alternative strategies for their automatic removal. We rejected the possibility of masking the reference sequences and/or the genome for simple repeats, because that could have decreased dramatically the sensitivity of the detection of TEs that have many simple repeats in their reference sequence. Moreover, this strategy does not guarantee the removal of simple repeats that are too degenerate from a regular pattern to be detected, but that could still produce spurious hits because of differences in simple repeat detection versus TE detection.

Instead, we settled on a two-step post-processing of our curated predictions that first identified all annotations that were less than a length threshold after removing regions that overlapped simple repeat regions. These putative spurious hits were then used as queries in a filtered BLAST against the BDGP TE reference set to “rescue” false spurious hits (i.e., real TEs) from true spurious hits. To develop this method, we used the *roo* family as a training set, for which we could easily partition spurious from real TE annotations. We tested the ability of three methods for simple repeat detection—RepeatMasker, Mreps, and TRF—to discriminate real from spurious *roo* annotations as a function of length remaining after simple repeat removal. We found that using RepeatMasker with a length threshold of 170 bp allowed all 236 spurious *roo* annotations to be identified with no real annotations identified as spurious (data not shown).

Using this threshold we detected 3,058 putative spurious hits, which were then searched with BLASTN (*E*-value > 1 × 10^−15^) using the “dust” filtering option against our reference TE sequence set. We found that only 18 of the 3,058 putative spurious hits were rescued as real annotations, indicating that our simple repeat filtering thresholds have high specificity. These 3,040 putative spurious hits were removed from the final set of Release 4 TE annotations submitted to FlyBase. Finally, to understand the source of these spurious hits in the auto-promoted TE models, we analyzed the overlap of the 3,040 spurious hits with Release 4 predictions generated individually by BLRn and RM. We find that 2,898 (95%) of the spurious hits overlapped a RM prediction, whereas only 1,255 (41%) of the spurious hits overlapped a BLRn prediction, indicating that RM generated a greater proportion of the spurious hits than BLRn.

## Discussion

We have developed and implemented a combined-evidence pipeline to annotate TEs in genome sequences and applied this novel system to detecting TEs in the Release 4 sequence of *D. melanogaster*. Our work fulfills the demand for a unified approach to TE annotation that capitalizes on the strength of multiple TE detection methods [3] and places TE annotation on common conceptual framework with gene annotation [5–9]. Compared with annotations generated for the Release 3 sequence [18], we confirmed precisely 743 out of 1,572 TE annotations. We adjusted the boundaries of 488, joined 80, changed the strand of 66, changed the name of 14, split 16, and described 4,573 new TE annotations. (Note that the number of modifications does not total 1,572 since multiple Release 3 elements were incorporated in a single join). These 4,573 new TE annotations are all supported by significant nucleotide homology to previously recognized families of TEs in *Drosophila*. According to our annotation the euchromatic TE fraction is now estimated to be 5.3% (cf. 3.86% in Release 3.1), and we found a substantially higher number of TEs (*n* = 6,013) than previously identified (*n* = 1,572). Most of the new TEs derive from small fragments of about a few hundred nucleotides long, and from highly abundant families not previously annotated (e.g., *INE-1*). Taking into account the heterochromatic TE fraction estimated by Hoskins et al. [25] and the fraction of this compartment (1/3 of the genome), we can estimate that in *D. melanogaster* TEs represent about 20% of the whole genome (about 5% of the euchromatin and about 50% of the heterochromatin). The pipeline allows rapid and thorough annotation of even the most complex TE models, including highly deleted and/or nested elements. We now estimate that 518 TE copies (8.6%) are inserted into at least one other TE, forming a nest. A detailed description of abundance and distribution of TEs in Release 4 based on the result of this annotation is in preparation. The full annotation is available through FlyBase (http://www.flybase.org) and the REPET database (http://dynagen.ijm.jussieu.fr/repet/).

### Performance

Our studies on the Release 3 sequence provide a first detailed genome-wide analysis of different methods for TE detection relative to a manually curated reference set of TE annotations. These results (see Tables 1 and 2) provide insight into the strengths and weaknesses of each method and therefore a deeper understanding of the consequences of algorithmic differences for TE detection. In general, our results suggest that BLRn can outperform RM with respect to the precise determination of TE boundaries, and that much of this improvement derives from the joining algorithm implemented in MATCHER. On the other hand, RM appears to be more sensitive for the detection of small and divergent TE copies. RM can detect small copies with less than 80% of identity with the reference sequence, while BLRn misses these small copies. This increase in sensitivity comes with a cost, as RM predicts many spurious hits for TE families with simple repeats in their reference sequence. Overall, we found that the differences between BLRn and RM make them very complementary for TE annotation when hits from both methods are chained with MATCHER, and that a simple-repeat-filtered version can be used to promote reliable TE models automatically.

There are many reasons why BLRn and RM perform differently. One obvious reason is that the initial word length used to seed the alignments is shorter for RM than for BLRn (nine for Cross_match versus 11 for BLASTN). Another reason is that RM chooses its scoring scheme (a match–mismatch matrix) according to the background percent guanine/cytosine composition. A third explanation could come from the final Smith–Waterman alignment performed by RM, allowing it to produce longer alignments in low identity regions. Likewise, in some particularly difficult cases where a genomic TE copy has a duplicated segment, BLRn gives a better annotation because it relies only on BLASTN hits that allow a small level of overlap between adjacent hits. The final Smith–Waterman alignment performed by RM is disturbed in these cases, at best placing a gap to face the duplicated segment. The first two reasons are a matter of parameter values, and the differences may simply be due to our use of default parameters. The more sensitive parameter set of RM has a cost in term of speed, and the trade-off between speed and sensitivity between BLRn and RM is not the same (BLRn is at least 3-fold faster). Using different parameter values could improve either BLRn sensitivity and/or RM speed. It remains to be determined to what degree the sensitivity of BLRn can be improved to a level equivalent to RM just by changing the BLASTN parameters, since the use of different match–mismatch matrices (each optimal for a background percent guanine/cytosine level) is an important difference between the two methods, and may limit BLRn sensitivity gains.

### Pitfalls

From our manual edits, we were able to identify some pitfalls that could be avoided in future attempts at a fully automated TE annotation process. One of the most important problems arises from the annotation of symmetrical structures, such as terminal inverted repeats (TIRs) and long terminal repeats (LTRs). There may be palindromic structures, such as in the *FB* element [26]. Often the two TIRs of a genomic *FB* element are detected on different strands, i.e., the 5′ TIR on the positive and the 3′ TIR on the negative strand. This happens because the two TIRs are not identical in the reference sequence. Thus, if the two TIRs of the genomic copy are more similar to each other than to the appropriate TIR in the reference sequence, only one TIR of the reference (the most similar one) is used to detect the two genomic TIRs, but on different strands. To avoid this type of manual edit, we suggest using a reference sequence with identical TIRs. A similar pitfall occurs with LTR retrotransposons. If the two LTRs are not identical on the reference, a genomic copy can be detected with two 5′ LTRs (or 3′ LTRs) if its LTRs are more similar to each other than to the appropriate LTRs of the reference sequence. If a join is necessary because of an indel in the genomic copy, our algorithm fails since the coordinates on the reference sequence are not collinear. To avoid this, we suggest using reference sequences with identical LTRs.

Some non-LTR retrotransposon genomic copies have to be extended in 3′ direction to encompass the entire polyadenine (poly[A]) tail. This occurs because the reference sequence has a shorter poly(A) tail than a particular genomic copy. In general, these cases are easily identified by observing an overlapping poly(A) simple repeat at the 3′ end of the element. One solution to this problem is to extend the poly(A) tail of non-LTR retrotransposons in the reference set to the length of the longest observed genomic copy.

The biggest pitfall we have encountered is the problem posed by simple repeats that exist in TE reference sequences. Without a specific treatment of this problem we would have included 3,040 spurious hits—approximately one-third of our original set of annotations. Filtering simple repeats on the genomic or reference sequences without affecting the sensitivity of TE detection is not easy. We have developed an effective (but ad hoc) two-step filtering strategy, but the magnitude of this problem leaves room for future improvements. Currently we employ RM to detect simple repeats, although refined parameter optimization may reveal that other more specialized simple repeat detection software, such as TRF [21], Mreps [22], or other methods (e.g., [27]) might be more appropriate. A careful evaluation of methods and parameters for simple repeat detection may allow us to decrease our 170-bp threshold and avoid the rescue step.

Regardless of the best method or criteria to detect simple repeats, the existence of simple repeats in TE reference sequences raises an important problem, since it is difficult to unambiguously determine whether a simple repeat with homology to a TE is a spurious hit or reflects a true remnant of that TE in the genome. Our methods guarantee that if we leave a spurious hit in the annotation because of homology with a simple repeat, it is more than 170 bp long. Moreover, any potentially real TE labeled as spurious that did not survive our rescue strategy bears no unique hallmarks of being generated by a TE. Nevertheless, the possibility of the involvement of TEs in the genesis of microsatellites [28] highlights the fundamental biological difficulty in resolving real from spurious simple repeats in a whole-genome TE annotation.

### Conclusions and Future Directions

We have shown in this work that a combined-evidence framework can improve the quality and confidence of TE annotations in *D. melanogaster*. Our automated pipeline allows us to annotate TEs on a genomic scale quickly and accurately, and the integration of our pipeline with the Apollo annotation tool also allows rapid evaluation and manual editing of TE annotations for even complex TE models. Based on the lessons learned in this study, we are continuing to develop and improve our pipeline. We are automating several classes of the manual edits that we have identified and expect that progressively fewer manual edits will be necessary in the future, allowing application of our pipeline to larger genome sequences such as the human sequence. One possible solution to the simple repeat problem is to develop a “combined sensor” model that would seek to resolve competing signals between simple repeats and TE models. It may also be possible to predict nested elements that require manual edits by using a stochastic context-free grammar [29] approach to model the different components of TE nests more generally; stochastic context-free grammars may also be useful in resolving problems encountered in annotating TEs with terminal repeats. The annotations presented here could be used as a training set to estimate the utility of these types of models.

We have observed several cases in the genome annotation where one or more de novo methods (RECON, BLRa, BLRtx, and TE-HMM) simultaneously support a potential sequence belonging to a new TE family. In addition, results of our analyses with tools that detect anonymous TEs (see Table 2) suggest that there may be many additional families of TEs yet to be discovered in the *D. melanogaster* genome. Since the methods that support these predictions potentially suffer from a high false positive rate, we have chosen not to include them in our current annotation, since more work needs to be done to validate these potential new TE families. Nevertheless the combined evidence for some of these elements is compelling and such cases are available for mining in our current results.

In general, the problem of TE discovery remains a major challenge for TE annotation. A good TE annotation relies critically on an expertly assembled reference sequence set, data that currently cannot be obtained in an automatic fashion. This crucial step is now the bottleneck in any method or pipeline to annotate TEs in genome sequences (see also [3]). The task to assemble such reference sets will be most difficult in genomes where only a few TE families are known. In these situations, we will need good de novo TE detection procedures [10–16] that can only be trained and evaluated properly using high-quality TE annotations in well-studied systems such as *Drosophila*. We hope that the TE annotations presented here will serve to further the development and refinement of TE discovery and annotation methods in general, as the Release 3.1 annotations have served for the development of our current methods.

Finally, we are also developing our pipeline to include methods for the detailed annotation of the structural features (open reading frames, LTRs, etc.) in TE sequences. Development of such detailed annotation methodologies will allow a detailed evaluation of the coding and expression potential of individual TE annotations in genomic sequences. Moreover, the ability to automatically annotate structural features of TEs will facilitate the manual curation and validation of candidate TE sequences resulting from one or several different de novo TE discovery methods [10–16]. Continued development of this pipeline, together with other advances in the field of TE genome informatics, will lead to a robust computational framework that can shed light on the origin and impact of TEs in modern genomes.

## Materials and Methods

### Data.

The *D. melanogaster* genomic sequences and TE reference sets are available from BDGP (http://www.fruitfly.org/). The Release 3.1 *D. melanogaster* genomic sequences and their TE annotations have been extracted from the GAME-XML files. The Release 4 *D. melanogaster* genomic sequences have been downloaded as fasta files. TE reference sequence sets v.7.1 (used by Kaminker et al. [18]) and v.9.0 have been downloaded from BDGP.

Sequences of the TEs were also obtained from the Repbase Update database release 8.12 [30], which contains all known repeated sequences including TEs (downloaded from http://www.girinst.org). We used them to detect unknown families by similarity with TEs from other species.

### Sequence analysis software.

We have improved three C++ programs: BLASTER, MATCHER, and GROUPER, previously presented in Quesneville et al. [13]. BLASTER can compare two sets of sequences: a query databank against a subject databank. For each sequence in the query databank, BLASTER launches one of the BLAST programs (BLASTN, TBLASTN, BLASTX, TBLASTX, BLASTP, or MegaBLAST) [17,31–33] to search the subject databank. Each BLAST search is launched in parallel on a computer cluster. BLASTER is not limited by the length of sequences. It cuts long sequences before launching BLAST and reassembles the results afterwards. Hence, it can work on whole genomes, in particular, to compare a genome with itself to detect repeats. The results of BLASTER can then be treated by the MATCHER and GROUPER programs described below. For the experiments conducted here, NCBI-BLAST2 (ftp://ftp.ncbi.nlm.nih.gov/blast/) programs were used with default parameters, using as a query genomic fragments of 50 kb, overlapping by 100 bp.

MATCHER has been developed to map match results onto query sequences by first filtering overlapping hits. When two matches overlap on the genomic (query) sequence, the one with the best alignment score is kept; the other is truncated so that only nonoverlapping regions remain on the match. As a result of this procedure a match is totally removed only if it is included in a longer one with a best score. All matches that have *E*-value greater than 1 × 10^−10^ or length of 20 or less are eliminated.

Long insertions (or deletions) in the query or subject could result in two matches, instead of one with a long gap. Thus, the remaining matches are chained by dynamic programming. A score is calculated by summing match scores and subtracting a gap penalty (0.05 times the gap length) and a mismatch penalty (0.2 times the mismatch length region) as in [34].

The chaining algorithm ([35], pp. 325–329) is modified to produce local alignments. A match is chained with a chain of other matches only if the resulting score is greater than the score of the match alone. Thus, the chaining is stopped if the score of the resulting chain of matches is less than if the match is not chained. The best-scoring chain is kept. Then to identify other match chains, the chain previously found is removed, and we search again for the next best match chain. This is done iteratively until no chain is found. This algorithm is repeated independently for match on strand +/+, +/−, and −/+. A maximum of 20% of overlap between the matches is allowed. The chaining algorithm allows the recovery of TEs containing long insertions, and therefore can identify nested elements accurately: they appear as a long insertion inside another TE.

GROUPER uses matches (or chained matches) to gather similar sequences into groups by simple link clustering. A match belongs to a group if one of the two matching sequence coordinates overlaps a sequence coordinate of this group by more than a given length coverage percentage threshold (a program parameter). If the two matches overlap with this constraint, their coordinates are merged, taking the extremum of the both. Groups that share sequence locations—not previously grouped because of a too low length coverage percentage—are regrouped into what we call a cluster. As a result of these procedures, each group contains sequences that are homogeneous in length. A given region may belong to several groups, but all of these groups belong to the same cluster.

RepeatMasker (http://www.repeatmasker.org) screens for TEs and low-complexity DNA sequences. It detects TEs in nucleic acid sequences by nucleic sequence alignment with previously characterized elements using the program Cross_match (http://www.phrap.org/phredphrapconsed.html) or WU-BLAST (http://blast.wustl.edu) with the script MaskerAid [36]. Both alignment programs perform their Smith–Waterman alignments by first identifying exact word matches and restricting the alignment to a band or matrix surrounding this exact match or matches. According to the background percent guanine/cytosine composition, different similarity matrices (each optimal for a background percent guanine/cytosine level) are used. RepeatMasker annotates the parts of sequences that are very similar to an element from a reference set of “known elements”. Low-complexity DNA regions are detected when stretches of nucleotides are GC- or AT-rich. Simple repeats are detected by searching all di- to pentameric and some hexameric repeats, allowing for possible variation within repeats.

RECON [10] is an automated process for de novo identification of new repeat sequence families in sequenced genomes. It searches genomic sequences for long repeats and clusters them in groups of similar sequences. TE copies from a given family are expected to cluster together. Its algorithm clusters repeats obtained by an all-by-all sequence comparison (here using BLASTER with BLASTN) and redefines the clusters by the aggregation of endpoints in a multiple alignment of the identified regions. In this way it tends to distinguish true TE copies from copies in a segmental duplication.

We have shown previously how base compositional differences can be used as a tool for detection and analysis of novel TE sequences [14]. Hidden Markov models are used to take into account the base composition of the sequences and the heterogeneity between coding and noncoding parts of sequences. We use three sets of sequences from *D. melanogaster* containing class I TEs, class II TEs, and cellular genes. Each of these sets has a distinct, homogeneous composition, enabling us to distinguish between the two classes of TEs and the genes. This approach can be used to detect and annotate TEs in genomic sequences and complements the current homology-based TE detection methods. Furthermore, the hidden Markov model method is able to identify the parts of a sequence in which the nucleotide composition resembles that of a coding region of a TE. This is useful for the detailed annotation of TE sequences, which may contain an ancient, highly diverged coding region that is no longer fully functional.

### Comparison of predictions and annotations.

We automatically compared predictions obtained with different computational methods to the Release 3.1 TE reference annotations in two ways, each implemented in a custom Python script.

The first calculated the nucleotide overlaps between the predictions and reference annotations, and computed the genome-wide sensitivity and the specificity. These values were obtained from equations (1) and (2) and the counts of true positive (TP—correctly annotated as belonging to a TE), false positive (FP—falsely predicted as belonging to a TE), true negative (TN—correctly annotated as not belonging to a TE), and false negative (FN—falsely predicted as not belonging to a TE) nucleotides.


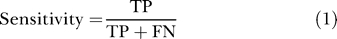



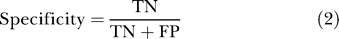


A high sensitivity indicates that a method misses few TE nucleotides (few false negatives). A high specificity indicates that a method finds few false positive nucleotides.

**Figure 3:**
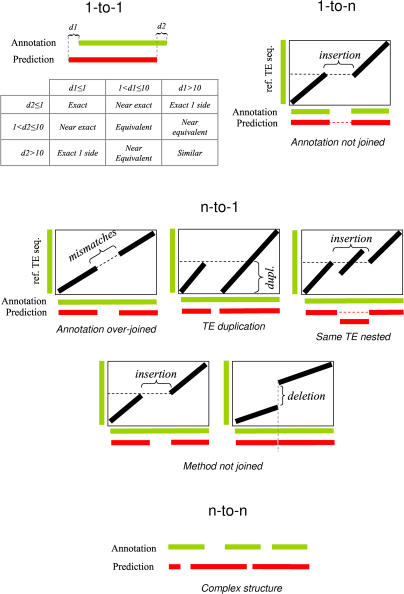
Categories of Possible Boundary Comparisons between Predictions and Reference Annotations The different cases taken into account can be grouped according to one-to-one (1-to-1), one-to-many (1-to-*n*), many-to-one (*n*-to-1), or many-to-many (*n*-to-*n*) relationships.

The second Python script compared the boundaries of predictions to the boundaries of the reference annotations. For each prediction under test, we searched the reference annotations that overlapped on the same genomic region. Different cases could be distinguished according to one-to-one, one-to-many, many-to-one, or many-to-many relationships (see Figure 3 for details).

For those that had a one-to-one correspondence with the same TE family, we calculated the difference in distances between predictions and annotations for their respective 5′ and 3′ coordinates. We categorized the differences in distance into three classes: ≤1 bp, ≤10 bp, or >10 bp. We called “exact” annotations those that had distances at both extremities ≤ 1 bp, “near exact” those for which the distance at one extremity was ≤ 1 bp and that of the other was >1 bp and ≤10 bp, and “one side exact” those for which one extremity was ≤1 bp and the other was >10 bp. Cases where both distances were > 1 bp and ≤ 10 bp were called “equivalent”; if one distance was > 1 bp and ≤ 10 bp and the other was >10 bp, the case was “near equivalent”; and if both distances were > 10 bp, the case was “similar”.

We also considered many-to-one relationships. Some were method errors in which a genomic copy (given by the reference annotation) had a large insertion or deletion. In this case, the two fragments (flanking the indel) were predicted as two separate copies, and the fragments were not joined. We called this error class “method not joined”. We also found cases in which two predictions were falsely considered as one in the reference annotation. Here, a long region of mismatch separated two fragments and the most parsimonious explanation was the independent insertion of two copies. These were “annotation over-joined” cases. We also found cases considered as one copy by the reference annotation, but that were in fact copies with a self-duplicated region. If the duplication was nested we call it “same TE nested”, or if not nested, “TE duplication”.

One-to-many relationships were cases in which two annotations in the reference were found joined by the method. We called this “annotation not joined”.

One-to-zero relationships corresponded to cases in which a prediction did not correspond to a reference annotation. “New TE” cases were copies identified by the method under test but not present in the reference annotation, and “different TE” cases were those overlapping a reference annotation but with a different TE family name. A TE prediction included in a prediction of a different family already involved in a given relationship with reference annotations, was called “new nest” if no corresponding reference annotation could be found. Annotation correspondence of the same TE family but on different strand was called “other strand” if the relationship was one-to-one; otherwise they were “new TE”.

Finally we had a “complex structure” case when the relation was many-to-many.

The script could be also used in an anonymous mode to test boundaries of de novo predictions that do not use a specific reference sequence. The information used for such comparisons is of poorer quality since we do not have alignment coordinates on the reference sequence (i.e., RECON and TE-HMM), which renders several categories meaningless (e.g., “different TE”, but also “new nest”, “other strand”, and “TE duplication”).

## Abbreviations

BDGP: Berkeley *Drosophila* Genome Project
BLRa: all-by-all genome comparison with BLASTER using BLASTN followed by chaining with MATCHER and grouping with GROUPER
BLRn: BLASTER using BLASTN followed by chaining with MATCHER
BLRtx: BLASTER using TBLASTX with the entire Repbase Update as the database, followed by chaining with MATCHER
BLRtxNoDros: BLASTER using TBLASTX with the Repbase Update with *Drosophila* TEs removed as the database, followed by chaining with MATCHER
LTR: long terminal repeat
poly(A): polyadenine
RM: RepeatMasker using default parameters
RMBLR: RepeatMasker-BLASTER
RMm: RepeatMasker using default parameters followed by chaining with MATCHER
TE: transposable element
TE-HMM: hidden Markov Model that detects TE coding sequences based on nucleotide composition
TIR: terminal inverted repeat
TRF: Tandem Repeat Finder

## References

[pcbi-0010022-b01] Lander ES, Linton LM, Birren B, Nusbaum C, Zody MC (2001). Initial sequencing and analysis of the human genome. Nature.

[pcbi-0010022-b02] Kidwell MG, Lisch DR (2001). Perspective: Transposable elements, parasitic DNA, and genome evolution. Evolution Int J Org Evolution.

[pcbi-0010022-b03] Juretic N, Bureau TE, Bruskiewich RM (2004). Transposable element annotation of the rice genome. Bioinformatics.

[pcbi-0010022-b04] Misra S, Crosby MA, Mungall CJ, Matthews BB, Campbell KS (2002). Annotation of the *Drosophila melanogaster* euchromatic genome: A systematic review. Genome Biol.

[pcbi-0010022-b05] Mungall CJ, Misra S, Berman BP, Carlson J, Frise E (2002). An integrated computational pipeline and database to support whole-genome sequence annotation. Genome Biol.

[pcbi-0010022-b06] Allen JE, Pertea M, Salzberg SL (2004). Computational gene prediction using multiple sources of evidence. Genome Res.

[pcbi-0010022-b07] Ding L, Sabo A, Berkowicz N, Meyer RR, Shotland Y (2004). EAnnot: A genome annotation tool using experimental evidence. Genome Res.

[pcbi-0010022-b08] Ashurst JL, Chen CK, Gilbert JG, Jekosch K, Keenan S (2005). The Vertebrate Genome Annotation (Vega) database. Nucleic Acids Res.

[pcbi-0010022-b09] Haas BJ, Wortman JR, Ronning CM, Hannick LI, Smith RK (2005). Complete reannotation of the *Arabidopsis* genome: Methods, tools, protocols and the final release. BMC Biol.

[pcbi-0010022-b10] Bao Z, Eddy SR (2002). Automated *de novo* identification of repeat sequence families in sequenced genomes. Genome Res.

[pcbi-0010022-b11] Biedler J, Tu Z (2003). Non-LTR retrotransposons in the African malaria mosquito, *Anopheles gambiae:* Unprecedented diversity and evidence of recent activity. Mol Biol Evol.

[pcbi-0010022-b12] McCarthy EM, McDonald JF (2003). LTR_STRUC: A novel search and identification program for LTR retrotransposons. Bioinformatics.

[pcbi-0010022-b13] Quesneville H, Nouaud D, Anxolabehere D (2003). Detection of new transposable element families in *Drosophila melanogaster* and *Anopheles gambiae* genomes. J Mol Evol.

[pcbi-0010022-b14] Andrieu O, Fiston AS, Anxolabehere D, Quesneville H (2004). Detection of transposable elements by their compositional bias. BMC Bioinformatics.

[pcbi-0010022-b15] Edgar RC, Myers EW (2005). PILER: Identification and classification of genomic repeats. Bioinformatics.

[pcbi-0010022-b16] Price AL, Jones NC, Pevzner PA (2005). De novo identification of repeat families in large genomes. Bioinformatics.

[pcbi-0010022-b17] Altschul SF, Gish W, Miller W, Myers EW, Lipman DJ (1990). Basic local alignment search tool. J Mol Biol.

[pcbi-0010022-b18] Kaminker JS, Bergman CM, Kronmiller B, Carlson J, Svirskas R (2002). The transposable elements of the *Drosophila melanogaster* euchromatin: A genomics perspective. Genome Biol.

[pcbi-0010022-b19] Lewis SE, Searle SM, Harris N, Gibson M, Lyer V (2002). Apollo: A sequence annotation editor. Genome Biol.

[pcbi-0010022-b20] Celniker SE, Wheeler DA, Kronmiller B, Carlson JW, Halpern A (2002). Finishing a whole genome shotgun sequence assembly: Release 3 of the *Drosophila* euchromatic genome sequence. Genome Biol.

[pcbi-0010022-b21] Benson G (1999). Tandem repeats finder: A program to analyze DNA sequences. Nucleic Acids Res.

[pcbi-0010022-b22] Kolpakov R, Bana G, Kucherov G (2003). mreps: Efficient and flexible detection of tandem repeats in DNA. Nucleic Acids Res.

[pcbi-0010022-b23] Locke J, Howard LT, Aippersbach N, Podemski L, Hodgetts RB (1999). The characterization of *DINE-1,* a short, interspersed repetitive element present on chromosome and in the centric heterochromatin of *Drosophila melanogaster*. Chromosoma.

[pcbi-0010022-b24] Meyerowitz EM, Hogness DS (1982). Molecular organization of a *Drosophila* puff site that responds to ecdysone. Cell.

[pcbi-0010022-b25] Hoskins RA, Smith CD, Carlson JW, Carvalho AB, Halpern A (2002). Heterochromatic sequences in a *Drosophila* whole-genome shotgun assembly. Genome Biol.

[pcbi-0010022-b26] Potter S, Truett M, Phillips M, Maher A (1980). Eucaryotic transposable genetic elements with inverted terminal repeats. Cell.

[pcbi-0010022-b27] Sagot MF, Myers EW (1998). Identifying satellites and periodic repetitions in biological sequences. J Comput Biol.

[pcbi-0010022-b28] Wilder J, Hollocher H (2001). Mobile elements and the genesis of microsatellites in dipterans. Mol Biol Evol.

[pcbi-0010022-b29] Durbin R, Eddy SR, Krogh A, Mitchison G (1999). Biological sequence analysis: Probabilistic models of proteins and nucleic acids.

[pcbi-0010022-b30] Jurka J (2000). Repbase update: A database and an electronic journal of repetitive elements. Trends Genet.

[pcbi-0010022-b31] Gish W, States DJ (1993). Identification of protein coding regions by database similarity search. Nat Genet.

[pcbi-0010022-b32] Altschul SF, Madden TL, Schaffer AA, Zhang J, Zhang Z (1997). Gapped BLAST and PSI-BLAST: A new generation of protein database search programs. Nucleic Acids Res.

[pcbi-0010022-b33] Zhang Z, Schwartz S, Wagner L, Miller W (2000). A greedy algorithm for aligning DNA sequences. J Comput Biol.

[pcbi-0010022-b34] Chao KM, Zhang J, Ostell J, Miller W (1995). A local alignment tool for very long DNA sequences. Comput Appl Biosci.

[pcbi-0010022-b35] Gusfield D (1997). Algorithms on strings, trees, and sequences: Computer science and computational biology.

[pcbi-0010022-b36] Bedell JA, Korf I, Gish W (2000). MaskerAid: A performance enhancement to RepeatMasker. Bioinformatics.

